# Effects of Pistacia Atlantica Extract on Erythrocyte Membrane Rigidity, Oxidative Stress, and Hepatotoxicity Induced by CCl_4_ in Rats

**DOI:** 10.5539/gjhs.v7n7p32

**Published:** 2015-03-26

**Authors:** Mohsen Tolooei, Ali Mirzaei

**Affiliations:** 1 Department of biology, Islamic Azad University, Ahar branch, Ahar, Iran; 2 Medicinal Plants Research Center, Yasuj University of Medical Sciences, Yasuj, Iran

**Keywords:** antioxidant, *Pistacia atlantica*, SOD, catalase, liver enzyme, lipid peroxidation

## Abstract

**Background:**

Previous findings have suggested that antioxidants may reduce the levels of free radicals, which induce oxidative damage and play a key role in various diseases. Thus, we evaluated the protective activity of a *Pistacia atlantica* extract on erythrocyte membrane rigidity, oxidative stress, and hepatotoxicity induced by carbon tetrachloride (CCl_4_) in rats.

**Materials and Methods:**

Fresh leaves of *P. atlantica* were collected from the mountains in Yasuj, Iran. Acute oral toxicity (LD_50_) was evaluated in Wistar rats (200–230 g). Animals were randomly divided into 4 groups, out of which the negative and plant control groups received distilled water and *P. atlantica* extracts (500 mg/kg), respectively. The toxic rat group received CCl_4_, while the treatment group received CCl_4_ + *P. atlantica* extract. Blood plasma was utilized for the estimation of enzyme markers and lipid peroxidation, whereas hemolysate was applied for the determination of superoxide dismutase (SOD) and catalase activities. The levels of cholesterol and phospholipids in erythrocyte membranes were also determined. Rats were killed under anesthesia by cervical dislocation; liver was isolated from each rat and tissues homogenization was prepared for biochemical parameters such as malondialdehyde (MDA) and reduced glutathione (GSH) levels.

**Results:**

LD_50_ values were determined for doses >3000 mg/kg (p.o.). The activities of glutamic pyruvate transaminase (GPT), glutamic oxaloacetate transaminase (GOT), alkaline phosphatase (ALP) and GSH in the protected group were significantly (p < 0.001) reduced compared with those of toxic rats. In addition, we observed a decrease in the cholesterol level and an increase in red blood cell membrane phospholipids, SOD, and catalase activities (p < 0.001) in the protected group, as compared with toxic rats. Administration of *Pistacia atlantica* extract normalized liver tissue MDA level (p < 0. 01) when compared to CCl_4_ treated group.

**Conclusion:**

The *P. atlantica* extract was able to normalize the levels of biochemical markers, including liver enzyme markers, first-line defense enzymes, and lipid peroxidation markers.

## 1. Introduction

Free radicals attack unsaturated fatty acids within the cell membrane and produce lipid peroxidation, which is responsible for heart disease, stroke, arteriosclerosis, cancer, as well as the aging process (Finkel et al., 2000). In the physiological state, the body is protected from free radical damage by enzymatic defense systems such as super oxide dismutase (SOD) and catalase, and also by non-enzymatic compounds supplied by endogenous antioxidants, including β-carotene, α-tocopherol, vitamin C, and uric acid (Ago et al., 2010). However, if the activity of endogenous antioxidant enzymes is insufficient to neutralize the free radicals, oxidative stress increases, resulting in a variety of chronic diseases, including diabetes mellitus, cancer, and cardiovascular disease (Lennon et al., 1991).

In contrast, in the pathological state, for example, in case of oxidative stress and lipid peroxidation, the cholesterol/phospholipid (C/P) ratio rises, resulting in an increase in the asymmetry, rigidity, and microviscosity of the erythrocyte membranes, and therefore a reduction in cell fluidity and deformability (Stubbs & Smith, (1984). The cholesterol and phospholipids in erythrocyte membranes are known to play an important role in membrane function, permeability, fluidity, and cell integrity (Fadeel & Xue, 2009).

The liver plays a key role in the biosynthetic and metabolic processes of the body. In particular, since the liver is an important organ for detoxification, it may be damaged by carbon tetrachloride (CCl_4_) intoxication. A previous study has shown that hepatic enzyme markers, including glutamic pyruvate transaminase (GPT), glutamic oxaloacetate transaminase (GOT), alkaline phosphatase (ALP), and lipid peroxidation, are increased in response to oxidative stress (Rahman, 2001). Medicinal plants play an important role in the prevention of hepatic diseases. Diverse varieties of the *Pistacia* genus grow in different provinces in Iran (Valipour, 1995), which is a major source of pistachios, including three basic varieties: *Pistacia vera, P. khinjuk*, and *P. atlantica* (Behboodi, 2003).

*P. atlantica* is considered a beneficial tree since it yields fruits and grows in many regions of Iran, including the Zagros Mountains. The resin and fruit oils of *P. atlantica* have traditionally been used in the treatment of gastrointestinal disorders and throat infections, and may have antibacterial, anti-inflammatory, antiviral, and antipyretic activities (Taran et al., 2010; Nabila et al., 2008). Pistachios are rich in phenolic substances, which are antioxidants that inhibit lipoperoxidation (Nabila et al., 2008). In this study, CCl_4_ was used to induce erythrocyte and hepatic cell damage (Parola et al., 1992), and hydroalcoholic *P. atlantica* extract was used to normalize the hepatic cell and erythrocyte cell membrane structures.

## 2. Materials and Methods

### 2.1 Plant Samples

Fresh leaves of *P. atlantica* were collected from the mountains in Yasuj, Iran. The identification of plant samples was confirmed by a taxonomist at the Department of Botany of Yazd University. Samples were washed, dried in the shade, and ground into a powder. Ground samples were extracted with ethanol (70%) and concentrated with a rotary evaporator (Heidolph Laborota 4000, Germany). The crude extract was subsequently concentrated and stored at −20°C until further analysis. A voucher specimen was deposited in the Medical Biochemistry laboratory for future reference (MPRC P.Ch.002/2006).

### 2.2 Experimental Design

This study was carried out on 4 groups of healthy male albino rats. The animals (weighing 200–230 g) were obtained from our rat colony and housed in a well-ventilated animal house at 22 ± 2 °C under conditions of 12-h dark/light cycles. All experimental rats had free access to drinking water and conventional laboratory rat food, supplied by Dam Pars Company, Iran. All animal experiments were performed according to the ethical guidelines of the Institutional Animal Ethics Committee, Iran.

### 2.3 Acute Toxicity

Acute toxicity (LD_50_) was estimated (p.o.) in 21 male rats divided into 7 groups. The rats matched for weight and size, and were fasted for 3 h before the investigation. The first group served as a control group and received a single dose of 1 ml/kg distilled water p.o. Groups 2–7 were treated with a single dose of hydroalcoholic *P. atlantica* extract (500–3000 mg/kg).

Housed animals were constantly inspected for abnormal behavior and symptoms of toxicity for 1 h after the treatment, and they were sporadically examined every 4 h for 1 day and every 24 h thereafter for a period of 14 days (Twaij et al., 1993). All experimental rats had free access to drinking water and standard laboratory rat diets. The LD_50_ value was determined by the arithmetical procedure of Karber (Turner, 1965).

### 2.4 Hepatic Toxicity and Erythrocyte Cell Membrane Studies

Animals were divided into 4 groups, each containing 6 rats. Distilled water was used as a carrier for the *P. atlantica* extract (0.5g/kg body weight/day p.o.). Olive oil was used as a carrier for CCl_4_ (1 ml/kg body weight p.o.), which was administered 3 times a week for 14 days. The following animal groups were used: group I (negative control), olive oil; group II (extract control), *P. atlantica* extract + olive oil; group III (toxic group), CCl_4_ + olive oil p.o.; and group IV (protected group), *P. atlantica* extract + CCl_4_ p.o.

All experimental rats were fasted for half a day and anesthetized with diethyl ether. Blood was obtained by heart puncture into tubes containing heparin as an anticoagulant.

Rats were killed under anesthesia by cervical dislocation; liver was isolated from each rat and prepared for tissues homogenization of biochemical parameters such as MDA and GSH levels.

After centrifugation of whole blood at 3000 rpm, plasma was collected. The buffy coat was removed, and the packed cells were washed 3 times with saline (0.9% NaCl). Packed cells were lysed, using cold distilled water, resulting in the formation of a hemolysate. The hemolyzed cells were subsequently centrifuged at 7000 rpm for 30 min to allow the erythrocyte membranes to settle at the bottom of the tubes.

Plasma was used for GPT, GOT, and ALP, which was analyzed with standard kits from local laboratories in Iran. The results were described in units/liter (U/l). Plasma was also used for the measurement of lipoperoxidation (Gutteridge, 1982), whereas the hemolysate was used to analyze the activities of SOD (Misra & Fridovich 1972) and catalase (Beers RF & Sizer., 1952).

Red blood cell membrane lipids were also extracted (Folch et al., 1957), and used to measure the C/P ratio (Searcy & Bergquist, 1960). For hepatotoxicity evaluation malondialdehyde (MDA) and reduced glutathione (GSH) were estimated in tissues homogenized (Ohkawa et al., 1979; Ellman, 1995).

### 2.5 Statistical Analysis

Comparisons between different groups were performed using analysis of variance (ANOVA) and post-hoc tests. The results were expressed as mean ± standard deviation (SD). *P*-values less than 0.05 were considered significant.

## 3. Results

No evidence of oral acute toxicity or mortality was observed in the different groups of rats after 14 days, even with the maximum dose of 3000 mg/kg body weight. Thus, the LD_50_ of extracts was calculated for doses >3000 mg/kg.

Treatment with the *P. atlantica* extract did not produce significant changes in the enzyme levels compared with the negative control (Table 1). However, there was a significant (P ≤ 0.05) increase, compared with the control group, in the hepatic levels of GPT, GOT, and ALP enzymes following CCl_4_ administration in toxic rats. Moreover, administration of the *P. atlantica* extract (500 mg/ml) to the protected group significantly lowered the level of CCl_4_-induced hepatic enzymes, compared with the toxic group.

**Table 1:** Results of *Pistacia atlantica* extract on Plasma hepatic markers ALT, AST and ALP in rats intoxicated by CCl_4_

Group	ALT (U/l)	AST (U/l)	ALP (U/l)
I	70.7±1.3	58.1±2.1	71.8±1.4
II	65.3±1.6	54.1±1.9	70.6±1.5
III	254±4.4^a^	232.8±8.5^a^	213.3±9.7^a^
IV	209.8±5^ab^	211.3±9.3^ab^	171.8±11^ab^

Values are means ± standard deviation for six rats per group;

A Statistically significant difference *vs*. Group I (*p* < 0.001).

b Statistically significant difference *vs*. Group III (*p* < 0.001).

Lipid peroxidation in the plasma and liver tissue were increased in rats that received CCl_4_. However, treatment with an oral dose of *P. atlantica* extract (500 mg/kg) significantly decreased the lipid peroxidation levels, compared with the control group (Table 2).

**Table 2:** Results of *Pistacia atlantica* extract on red blood cell antioxidant enzymes and lipid peroxidation products in rats intoxicated by CCl_4_

Group	Lipid peroxidation × 10^-6^ (units)	Enzyme activities (units/mg protein)	
		SOD	Catalase
I	0.24±0.03	182.6±10.5	1.46±0.14
II	0.21±0.01	185.4±3.4	1.5±0.18
III	0.51±0.02^a^	73.1±4^a^	.39±.08^a^
IV	0.40±0.05^ab^	99.5±8.5^ab^	0.78±0.1^ab^

Values are means ± standard deviation for six rats per group;

a Statistically significant difference *vs*. Group I (*p* < 0.001).

b Statistically significant difference *vs*. Group III (*p* < 0.001).

Intoxication of rats with CCl_4_ produced a significant decrease in the hepatic tissue MDA, SOD and catalase activities, whereas the activity of these enzymes increased in the protected group (P ≤ 0.05) (Table 2).

In addition, administration of CCl_4_ in toxic rats brought about an increase in the level of membrane cholesterol and a reduction in the membrane phospholipids. However, treatment with the *P. atlantica* extract prevented cell membrane changes by decreasing the cholesterol concentration and increasing membrane phospholipid levels (Table 3).

**Table 3:** Results of *Pistacia atlantica* extract on red blood cell membrane lipids in rats intoxicated by CCl_4_

Group	Cholesterol (mg/100μl)	Phospholipid (mg/100μl)	Cholesterol/Phospholipid
I	0.53±0.01	1.15±0.02	0.47±0.02
II	0.49±0.01	1.15±0.04	0.43±0.03
III	1.09±0.14^a^	0.73±0.05^a^	1.5±0.06^a^
IV	0.82±0.12^ac^	0.84±0.03^ac^	0.98±0.17^ab^

Values are means ± standard deviation for six rats per group;

a Statistically significant difference *vs*. Group I (*p* < 0.001).

b Statistically significant difference *vs*. Group III (*p* < 0.001).

c Statistically significant difference *vs*. Group III (*p* < 0.01).

CCl_4_ treated group had significantly increased MDA and decrease GSH contents (p < 0.001) in liver tissue compare to control group. Administration of *Pistacia atlantica* extract normalized liver tissue MDA level (p < 0. 01) when compared to CCl_4_ treated group. However, simultaneous treatment with *Pistacia atlantica* extract provided a significant increase in liver GSH levels (p < 0. 01) (Table 4).

**Table 4:** The effects of Pistacia *atlantica* extract on hepatic MDA and GSH contents in rats intoxicated by CCl_4_

Group	MDA(nmol/g protein	GSH (nmol/g tissue
I	63±3.3	11±0.2
II	59±4.1	12.6±1.3
III	131±5.4^a^	5.2±0.6^a^
IV	89.2±6.3^ac^	9.9±0.67^abb^

Values are means ± standard deviation for six rats per group;

a Statistically significant difference *vs*. Group I (*p* < 0.001).

b Statistically significant difference *vs*. Group III (*p* < 0.001).

c Statistically significant difference *vs*. Group III (*p* < 0.01).

MDA: Malondialdehyde, GSH: reduced glutathione.

## 4. Discussion

This study demonstrated the effectiveness of *P. atlantica* extract against oxidative stress, erythrocyte membrane rigidity, and hepatotoxicity. LD50 of the hydro-alcoholic extract of the *P. atlantica* was found to be greater than 3000mg/kg, which may be accepted as safe. Thus, the *P. atlantica* extract could be considered safe and non-toxic (OECD, 2001).

Lipid peroxidation in the physiological state in cells and tissues is low, however, increases in response to oxidative stress due to cell damage. Moreover, the levels of first-line defense antioxidant enzymes, including catalase and SOD, are normally decreased by oxidative stress.

Trichloromethyl as a peroxyl radicals, peroxidation of lipids, tissue damage, and liver injury were produced in hepatotoxic rats which treated with CCL_4_ (Lee, 2004). In addition, the liver marker enzyme, including GPT, GOT, ALP and MDA level has been shown to increase under conditions of hepatotoxicity, which may be followed by hyperbilirubinemia in severe cases (Rahman et al., 2001).

Furthermore, treatment with the *P. atlantica* extract decreased the activities of serum enzymes and MDA marker, indicating that this extract could normalize liver function.

The findings of the current study also confirmed CCl_4_-induced damage to red blood cells, which was demonstrated by the elevation of lipid peroxidation products, decreased catalase and SOD activities, and by the decreased fluidity of erythrocyte membranes.

Moreover, our study confirmed previously published results (Ojo et al., 2006) and showed that administration of the *P. atlantica* extract reduces lipid peroxidation and protects red blood cells against CCl_4_-induced damage.

SOD is a primary intracellular antioxidant enzyme that protects tissues and reduces cell damage induced by superoxide radicals. In addition, catalase is a hemoprotein antioxidant enzyme that rapidly converts superoxide radicals into water (Otitoju & Onwurah, 2006). The decrease in SOD and catalase activities, as well as elevated lipid peroxidation in CCl_4_-intoxicated rats may result from oxidative stress and/or decreased antioxidant defense (Mahdi et al., 1996).

Furthermore, this study revealed that *P. atlantica* extracts are able to improve free radical scavenging activity, resulting in increased SOD, catalase activities and reduced glutathione level and normalized of lipid peroxidation.

Glutathione is an antioxidant indicator with protective function in the metabolism of many toxic agents. It acts as a free radical scavenging agent and conserves cytochrome P-450 by inhibited of lipid peroxidation (Yuan *et al.*, 2008).

In this research, *P. atlantica* extract significantly increased and maintain the liver GSH potential when compared to CCl4- treated rats group. The mechanism of protection by *P. atlantica* extract against CCl4 toxicity may be due to restoration of the GSH content.

Lipid peroxidation is one of the most important outcomes of free-radical damage to tissues. Peroxidation of fatty acyl groups generally occurs in membrane phospholipids and may change the physicochemical characteristics of membrane lipid bilayers (Lee, 2004). The findings of the present study showed that treatment of rats with CCl_4_ induced peroxidation of red blood cells, which may have resulted in hemolytic changes. We also observed a positive correlation between membrane oxidation and the C/P ratio (McConnell & Hubbell, 1971).

However, treatment of experimental animals with the *P. atlantica* extract prevented the alteration of membrane fluidity and decreased the C/P ratio. Thus, the *P. atlantica* extract participated in peroxidation by inhibiting free-radical damage to biological membranes (Cooper et al., 1977).

In this study, a similar pattern was observed in the activity of enzymes and lipid peroxidation in rats. Moreover, the reversal in enzyme liver marker activity after treatment with the *P. atlantica* extract may have resulted from decreased oxidative levels. The *P. atlantica* extract may be able to directly neutralize reactive oxygen metabolites because of the presence of different antioxidant substances or increase the synthesis of antioxidant molecules/enzymes, which revealed by increased in reduced glutathione level in rats (Gupta et al., 2002).

Application of *P. atlantica* in traditional medicine for the treatment of jaundice and hypertension may be due to its antioxidant activity (Bentley & Trimen, 1980). The hepatotoprotective potential of the *P. atlantica* extract may result from its antioxidant property (Gutteridge, 1982) and its ability to stabilize membranes (Meena et al., 2008) by inhibited of lipid peroxidation (Nabila et al., 2008).

## 5. Conclusion

Treatment with a *P. atlantica* extract normalized the levels of biochemical markers. In particular, *P. atlantica* demonstrated hepatoprotective activity by protecting against hepatic injury produced by CCl_4_. It is likely that the efficacy of *P. atlantica* is due to its ability to inhibit free radicals.
